# Situational Awareness Prediction for Remote Tower Controllers Based on Eye-Tracking and Heart Rate Variability Data

**DOI:** 10.3390/s25072052

**Published:** 2025-03-25

**Authors:** Weijun Pan, Ruihan Liang, Yuhao Wang, Dajiang Song, Zirui Yin

**Affiliations:** 1 Flight Technology and Flight Safety Research Base of the Civil Aviation Administration of China, Civil Aviation Flight University of China, Guanghan 618307, China; wjpan@cafuc.edu.cn (W.P.); 17726549663@163.com (D.S.); 2 College of Air Traffic Management, Civil Aviation Flight University of China, Guanghan 618307, China; wangyuhao496@gmail.com; 3 School of Transportation and Logistics, Southwest Jiaotong University, Chengdu 611756, China; yin@my.swjtu.edu.cn

**Keywords:** situational awareness, remote tower, air traffic controller, LightGBM, SHAP, eye tracking, HRV

## Abstract

**Highlights:**

**What are the main findings?**

- A TPE-optimized LightGBM model, trained with eye-tracking and heart rate variability data, effectively predicts situational awareness (SA) in remote tower controllers, achieving an RMSE of 0.0909, MAE of 0.0730, and an adjusted R^2^ of 0.7845.
- Feature importance analysis via SHAP revealed that eye-tracking behaviors and HRV features, such as gaze entropy and the ratio of low-frequency to high-frequency power, significantly influence the prediction of SA.

**What is the implication of the main finding?**

- The proposed model offers a robust, non-invasive approach for real-time SA prediction, providing valuable insights into controller performance in remote tower environments.
- This methodology can be extended to improve the safety and efficiency of remote tower operations by monitoring and intervening in controllers’ SA levels in real time.

**Abstract:**

Remote tower technology is an important development direction for air traffic control to reduce the construction and operation costs of small or remote airports. However, its digital and virtualized working environment poses new challenges to controllers’ situational awareness (SA). In this study, a dataset is constructed by collecting eye-tracking (ET) and heart rate variability (HRV) data from participants in a remote tower simulation control experiment. At the same time, probe questions are designed that correspond to the SA hierarchy in conjunction with the remote tower control task flow, and the dataset is annotated using the scenario presentation assessment method (SPAM). The annotated dataset containing 25 ET and HRV features is trained using the LightGBM model optimized by a Tree-structured Parzen Estimator, and feature selection and model interpretation are performed using the SHapley Additive exPlanations (SHAP) analysis. The results show that the TPE-LightGBM model exhibits excellent prediction capability, obtaining an RMSE, MAE and adjusted R^2^ of 0.0909, 0.0730 and 0.7845, respectively. This study presents an effective method for assessing and predicting controllers’ SA in remote tower environments. It further provides a theoretical basis for understanding the effect of the physiological state of remote tower controllers on their SA.

## 1. Introduction

The remote tower is a new technology solution for providing airport tower control (ATC) services. It replaces the direct field-of-view observations from a traditional tower with visual data. These data are provided by cameras through a series of sensors mounted on the surface of the airfield and a display located at the center of the remote tower [1]. Remote towers use digital and intelligent means to enable remote management of airports, freeing controllers from the traditional physical tower [2]. Remote tower technology is one of the key objectives in the International Civil Aviation Organization’s (ICAO) development plan for the next ten years. Its aim is to provide an innovative solution to address the cost of building and operating airports in harsh natural environments, where the visibility may be poor or the traffic may be sparse [3]. However, the emergence and proliferation of remote tower technology have led to changes in the controller’s work environment, air traffic information access and operational procedures, which pose new challenges to the controller’s situational awareness (SA) [4].

The theoretical foundations of SA are derived from cognitive science and human factors engineering. The most widely used SA model in air traffic management and other complex systems is Endsley’s proposed three-layer SA model [5], which defines three key SA levels: perception of elements in the environment (Level 1), comprehension of current situation (Level 2), and projection of future status (Level 3). SA directly influences a controller’s ability to accurately perceive, comprehend, and make decisions based on dynamic information, serving as the foundation for the successful execution of complex and dynamic tasks. Cognitive bias or potential risk can be identified at an early stage by real-time tracking and analysis of the changes in SA. This identification allows for timely intervention, which has great significance in ensuring the safety of remote tower aviation operations.

The most common standardized methods used to study the SA aviation assessment are freeze probe techniques, such as the SA global assessment technique (SAGAT) and SA of en-route air traffic controllers in the context of automation (SALSA) [5,6]. These techniques involve a complete freezing of current air control tasks during experiments, where a set of questions are answered based on the participants’ knowledge and understanding of the current environment. The SA scores are derived by recording the participants’ responses and comparing them to the current state of the actual environment. For example, Feng et al. used the SAGAT to classify pilots’ high and low SA groups and combined the results with EEG characterization. The authors found that the SAGAT scores could significantly differentiate between the high and low SA groups and achieved high classification accuracy using sensitive EEG features [7]. However, this method is highly intrusive and significantly disrupts task performance due to the freeze probe technique. Another common method is the SA rating technique (SART), which assesses the SA using a subjective questionnaire filled out by participants after a task is performed. This technique quantitatively measures the participants’ SA in three main dimensions: perceived need, resource availability, and information comprehension [8]. However, the post-test self-rating technique suffers from the problem of “people don’t always realize what they don’t know [9].” The subjective nature of participants’ self-assessment may cause uncertainty in the assessment of the existing state of situational awareness. Therefore, an objective and non-invasive method to assess the SA is urgently needed.

Several existing studies and applications have confirmed the use of eye movements as an effective and non-invasive real-time performance indicator of human visual attention and mental state [10,11,12]. Eye-tracking techniques have been widely utilized to study eye movements and improve safety and human performance in aviation activities [13,14]. Eye-tracking technology captures eye movements, including gaze, saccades, and pupil diameter, to provide a range of visual behavioral metrics [15,16]. These indicators have considerable importance in all three stages of SA. In the perception layer of SA, gaze behavior can reflect an individual’s ability to perceive critical information in the environment. For example, Li et al. verified that gaze behavior analysis could be used to enhance pilots’ decision-making efficiency and SA at mission-critical moments by evaluating the number of gazes and gaze duration [17]. At the comprehension layer, pupil size, gaze duration in specific regions, and gaze entropy can all be used to infer an individual’s processing load for complex information [18,19,20]. Satyajit et al. tested the sensitivity and reliability of eye-tracking measures to discern changes in task difficulty in a physical human–robot collaboration task, which involved an industrial robot used for object manipulation. The authors showed that resting gaze entropy and pupil diameter were the most reliable and sensitive workload measures to differentiate changes in task difficulty and learning [21]. At the projection layer, gaze sequences provide insights into participants’ attentional allocation patterns and strategic approaches when predicting future task states [22]. According to Bruder and Hasse, experts showed that more efficient allocation of attention and more flexible scanning strategies can be obtained by recording gaze sequences at different stages of a surveillance task, i.e., orientation, prediction, detection and review [23].

In addition, eye movement data have been used to assess factors such as fatigue and stress associated with SA. For example, Xue et al. found that, in addition to a decrease in pupil diameter, a decrease in sweep amplitude can indicate increased fatigue [24]. Moreover, Allsop and Gray and Vine et al. found that emergency-induced anxiety caused a higher visual scan entropy in non-military pilots [25,26].

In addition, heart rate variability (HRV) is widely used to assess cognitive workload. Hughes et al. demonstrated that HRV variations in high- and low-frequency ranges significantly reflect task complexity and duration [27]. Cognitive workload represents the mental effort required to process tasks, while SA involves perceiving, understanding, and projecting critical information for decision making. Cognitive workload and SA, while often considered distinct constructs, exhibit a complex and dynamic relationship. This relationship has been further explored in studies such as Mehta et al., who utilized HRV to examine SA fluctuations in offshore well control scenarios, finding that higher cognitive load was associated with reduced SA [28]. Similarly, Tang et al. combined HRV and subjective ratings to evaluate cockpit display interfaces, showing that optimized designs led to lower cognitive workload and enhanced SA [29]. These studies highlight HRV’s potential as an indirect measure of SA, supporting its application in improving SA assessment.

However, given the complex and task-dependent nature of the relationship between cognitive workload and SA, HRV alone may not fully capture the nuances of SA dynamics. Integrating HRV with eye-tracking data provides a more comprehensive assessment by combining physiological indicators of workload with real-time measures of attentional allocation.

Although SA research has made great progress in the aviation field, existing SA studies have primarily focused on pilots or area controllers, with relatively limited research on apron controllers, particularly in the context of remote towers. Since individuals need to perceive and understand relevant environmental elements, the patterns and criteria of SA will vary according to different environments and tasks [5]. Meanwhile, remote tower technology may introduce additional challenges to controllers’ situational awareness, such as the presentation of multiple streams of virtualized information and reduced familiarity with airport terrain due to the lack of direct out-the-window views traditionally available in physical towers, among other related issues. These factors highlight an urgent need for a non-intrusive and real-time method to monitor the SA of remote tower controllers, in order to ensure the safe operation of air traffic. Therefore, it is necessary to systematically study SA prediction for controllers performing control tasks under the scenario of a remote tower. Building on this need, this study aims to explore how HRV and eye-tracking data can enhance the monitoring of SA in remote tower controllers. Specifically, we focus on the following:

- Whether eye-tracking and HRV data can be combined with machine learning to construct a high-precision SA prediction model;
- Whether the model’s predictions can support the study and monitoring of SA in remote tower controllers.

The ultimate goal of this research is to develop an SA monitoring method that can be integrated into remote tower systems to optimize information presentation and workload management for controllers. In this study, we experimentally collect eye-tracking and HRV data in a simulated remote tower scenario. We insert probe questions that are designed based on the flow of the control task in the remote tower scenario and its corresponding SA hierarchy in a process-specific triggering manner in the control experiment task. The SA is directly calculated using the situation present assessment method (SPAM) in conjunction with the participants’ correct response rate and reaction time [30]. Subsequently, a LightGBM model optimized by a Tree-structured Parzen Estimator (TPE-LightGBM) is trained on a labeled dataset containing 25 eye-tracking and HRV features. The TPE-LightGBM is an improved light gradient-boosting machine (LightGBM) model based on the Tree-structured Parzen Estimator (TPE) method that automatically optimizes the hyperparameters. Its prototype LightGBM is an efficient machine learning model based on the gradient-boosting decision tree (GBDT), which is widely used in regression problems with multimodal data inputs due to its fast training speed, low memory footprint, ability to handle high-dimensional data, and excellent prediction performance [31,32,33,34,35]. In addition, we use the SHapley Additive exPlanations (SHAP) for model input feature selection and explain the effect of features on the SA through this game theory-based feature importance interpretation method [35]. In summary, the novel contributions of this study are as follows:

- Eye-tracking and heart rate variability data are experimentally collected, and the sample SAs are labeled using an improved SPAM method based on the remote tower controller task.
- A TPE-optimized LightGBM model is proposed for predicting remote tower controller SA based on eye-tracking and HRV data. It is combined with the SHAP for feature selection, providing an innovative and effective solution for the assessment and prediction of remote tower controller SA.
- The model is interpreted using the SHAP, providing a theoretical basis for understanding the effect of the physiological state of remote tower controllers on their SA.

## 2. Experiments

### 2.1. Design

The background of the simulated remote tower control experiment consists of the virtual airport “Hansha Airport”, which is designed based on Wuhan Tianhe Airport (ZHHH) and Changsha Huanghua Airport (ZGHA). The experimental meteorological conditions are set as daytime with clear and cloudless weather. Combined with the actual operation characteristics of the remote tower, the task design includes the following three scenarios:

- Scenario I: Three inbound and four outbound aircraft. At any given moment, no more than two aircraft are on the taxiway, resulting in relatively low ground traffic pressure. This task is relatively simple and close to the daily operation of the remote tower.
- Scenario II: Seven inbound and seven outbound aircraft. At peak times, up to five aircraft are on the taxiway, leading to increased ground traffic pressure. This scenario is consistent with the increase in traffic after a long period of remote tower operation.
- Scenario III: Three inbound and four outbound aircraft. During the control task, an aircraft is suddenly stranded on the taxiway after developing a technical problem, causing a traffic jam. The controller must adjust the taxiway path to avoid conflicts and reserve a taxiway for towing the faulty aircraft. To maintain a realistic scenario distribution while ensuring sufficient data for analysis, this scenario was presented less frequently in each set of experiments.

Prior to the start of the experiment, participants were informed of the experimental procedures, completed a practice session, and signed a consent form. Five minutes before the experiment, it was confirmed that the participants were wearing the physiological data acquisition equipment, but these data are not used in the subsequent analysis. When the experiment starts, the participants maintain the control posture, and the three monitors are synchronized to show the simulated tower exterior, including aircraft, runway, taxiway and parking space. The captain communicating with the participants was played by the experimenter. The captain interacted with the air traffic controllers through a communication system in a separate room, simulating a radio communication scenario. Each participant completes two sets of experiments for a total of 10 test scenarios, including four Scenarios I, four Scenarios II, and two Scenarios III. Flight sequences and flight numbers are randomized within the same type of scenarios. There is a five-minute break between each scenario and a gap of one day between each set of experiments. The first set of experiments started at 9:00 a.m., while the second set began at 3:00 p.m.

Each scenario is designed to be queried with two probe questions. The probe questions are inserted during each task run as process-specific triggers at critical time points of the task to avoid causing excessive disruption to the participant’s task rhythm. Examples of these time points include the time instance when a specific flight leaves the stand or when control is handed over. In the same scenario, traffic fluctuations remain relatively stable, and multiple simulation runs were conducted prior to data collection to verify the consistency of traffic patterns, aiming to minimize variability in traffic flow as much as possible. First, the experimenter asks the participant if he/she is ready to answer the probe question. After receiving confirmation from the participant, the points in time at which the query is sent and the participant replies with the confirmation are recorded in the experimental system. Subsequently, the experimenter asks the participant a specific probe question, to which the correct response is either “yes/no” or a number. The experimental system records the time taken to ask and answer each probe question, and the participant is required to answer each question within a time limit of 15 s. A set of experimental procedures last approximately 1.6 h.

### 2.2. Program

**Figure 1:**
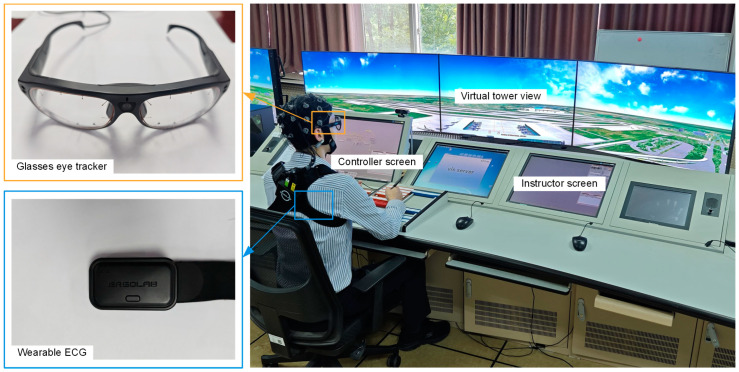
Experimental setting. The Instructor Screen is primarily used for developing arrival and departure plans and selecting experimental scenarios. The experiments are also synchronized with the EEG data collection, but the focus of the analysis in this study is only on the eye-tracking data and the HRV data.

We use the Tower Client simulator to reproduce interactions resembling those in a real remote tower operating environment. Its main interaction format and aircraft performance simulate the real remote tower controller work environment, where the participants are provided access ramp and runway-related visual information through a wide-view screen. At the same time, the simulator provides instructor-controlled and standardized scenarios with real-time display of timed information about the participant’s actions and ramp status, including synchronized controller and captain screen. A keyboard and a mouse can be used to perform major actions, such as initiating thrust, selecting taxiway/runway paths, and performing takeoffs/landings. Figure 1 shows the experimental setting.

We collect three types of information during the experiment: logs from the Tower Client simulator, eye-tracking data using a Tobii Pro Glasses 3 spectacle-based eye tracker with a sampling frequency of 100 Hz, and HRV data using an ErgoLAB Biosensing wearable electrocardiograph with a sampling frequency of 512 Hz. We also record the participants’ responses and reaction times to probe questions. The experimental data are divided based on the time points between the SA probes, where the probe closest to the time point of the current data is used as the labeled value for that set of data. At the end of the experiment, a total of 720 sets of data are obtained, each having a duration between 12 and 15 min.

The participants in this study were screened from the Air Traffic Management College of the Civil Aviation Flight University of China (CAFUC) to ensure that all participants met the Class I physical examination qualification standards defined in the Administrative Rules for Civil Aviation Personnel Physical Examination Qualification Certificates issued by the Civil Aviation Administration of China (CAAC). Additionally, the screening process ensured that all participants had normal vision, with no myopia or other visual impairments, to prevent any potential influence of vision-related factors on the experimental results. A total of 36 participants were chosen, including six experienced control instructors having 9000 ± 500 control hours and 30 control trainees who had completed the control training tasks and passed the corresponding examinations with experience of 500 ± 100 control hours.

This research complied with the China Ethical Review for Teaching/Research Involving Human Participants and was also approved by the Ethics Committee of the Civil Aviation Flight University of China. All participants were informed of this study’s objectives, procedures, potential risks, and benefits before participation. Written informed consent was obtained from each participant prior to the experiment. The confidentiality and anonymity of participants were maintained throughout the study.

## 3. Methods

### 3.1. Preprocessing and Feature Engineering

We import the collected raw eye-tracking (ET) data into the ErgoLAB data processing platform and extract features based on eye movement indicator thresholds reported in previous studies, combined with empirical values derived from our experimental observations [36,37,38,39,40,41,42]. The data are passed through a sliding median filter and processed using interpolation with a maximum gap length of 75 ms. Subsequently, the eye movement data are aligned with the timestamps of task events. For gaze detection, we set the minimum gaze duration to 60 ms and merge neighboring gazes that either have a gaze duration of less than 75 ms or a maximum gaze angle of less than 0.5°. Next, we set the duration of the saccade in a range of 10 ms to 200 ms using 2 pixels/ms as the minimum velocity threshold. Finally, we detect eye blinks based on the principle of pupil occlusion, where a blink is defined as a temporary loss of pupil visibility in both eyes. We linearly interpolate the pupil region, convert the pupil diameter to a minimum value of 2 mm, and use a threshold range of 70–350 ms to identify eye-blinking events.

**Figure 2:**
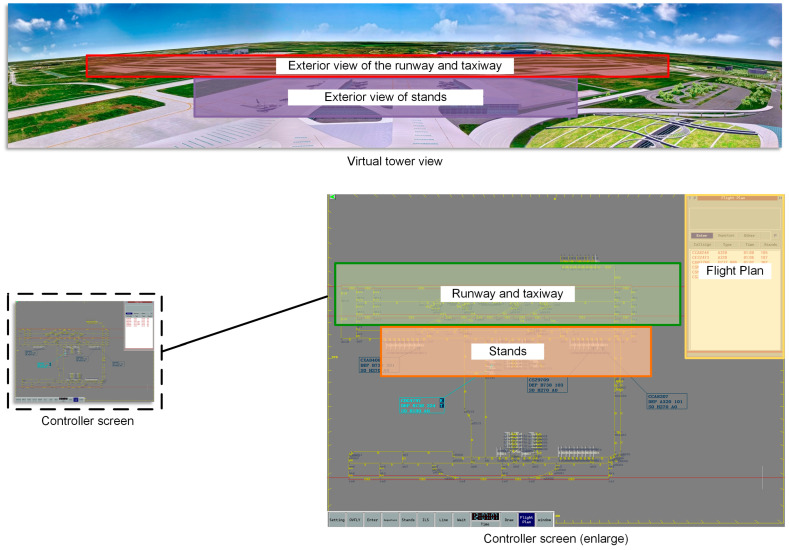
Schematic diagram of AOI area division.

Further, we analyze the gaze process using the Area of Interest (AOI) analysis, where the display is divided into five AOI regions based on the functionality and importance of the display so that the controller’s allocation of visual attention can be assessed more accurately. Figure 2 shows the layout of the AOIs.

Let the entire AOI region of the analog control system interface be defined by a set S={RTES={AOI1,AOI2…AOI5}. An AOI gaze sequence can be obtained by collecting visual gaze data over a certain time duration. Statistically, the proportion of gaze points appearing in these AOI regions $\left\{p_{\mathrm{AOI}1},p_{\mathrm{AOI}2},\ldots ,p_{\mathrm{AOI}5}\right\}$ can be calculated using the stationary gaze entropy (SGE) based on the Shannon’s entropy principle and the probability distribution of gaze coordinates. It measures the breadth and uniformity of the spatial distribution of gaze points [43] and is given in Equation (1) as follows:

(1) $$\mathrm{SGE}=-\left(\left(p_{AOI1}\log_{2}p_{AOI1}\right)+\left(p_{AOI2}\log_{2}p_{AOI2}\right)+\cdots +\left(p_{AOI5}\log_{2}p_{AOI5}\right)\right),$$

Another important type of information entropy is the gaze transition entropy (GTE), which can measure the degree of organization and regularity of gaze behavior by analyzing the probability distribution of gaze point transitions from one AOI region to another [43]. It can be calculated according to the following expression:

(2) $$\mathrm{GTE}=-\sum_{i=1}^{n}p_{i}(\sum_{j=1}^{n}p_{\mathrm{ij}}\log_{2}p_{\mathrm{ij}}),\;i\neq j,$$

In Equation (2), i and j denote the AOI regions that are being watched at the start of the jump and at the end of the jump, respectively. Furthermore, $p_{i}$ is the visual gaze appearing on the ith region, and $p_{\mathrm{ij}}$ is the proportion of visual gaze shifting from the ith region to the jth region as a proportion of all jumps in this segment of visual gaze data.

In addition, the HRV data are obtained from the acquired ECG data after feature extraction. First, the raw ECG data are preprocessed by MATLAB R2023a with an adaptive filter and the Bior4.4 wavelet transform to remove the power frequency interference and baseline drift. The bad channels are processed by averaging and interpolating the neighboring channels. Next, the ECG data are decomposed using the independent component analysis (ICA), and the artifactual components are manually removed.

Cardiac-related events are automatically detected from the preprocessed ECG signals using MATLAB extension HEPLAB with a maximum heart rate of 120 bpm and an R-Peak threshold of 70%. The time difference between neighboring R waves is calculated based on the timestamps of the acquired R waves to form an RR intervals sequence for obtaining the time-domain characteristics of the HRV. Subsequently, the RR interval sequence is analyzed to obtain the frequency spectrum using the fast Fourier transform (FFT) to further obtain the frequency domain characteristics of HRV.

Table 1 summarizes all the features used as inputs to the machine learning model, of which the first 19 correspond to eye-tracking-related features and the rest are HRV-related features. Fixation count and saccade count exhibit a relatively strong correlation (r = 0.82) compared to other features, indicating potential redundancy. However, the fixation count is associated with prolonged visual attention to specific areas, while the saccade count reflects shifts in attentional allocation. Removing either feature may impair the model’s ability to distinguish between focused and exploratory viewing behaviors. Therefore, both features were retained during the initial feature selection stage.

### 3.2. SPAM

We use the situation present assessment method (SPAM) to obtain direct SA measurements from participants [30]. It is a real-time SA assessment method, which aims to measure the participants’ ability to perceive, understand and predict the task environment by probing them with questions relating to the situation present while performing a task. Compared with other methods, SPAM is highly real time and objective and can effectively avoid recall bias.

**Figure 3:**
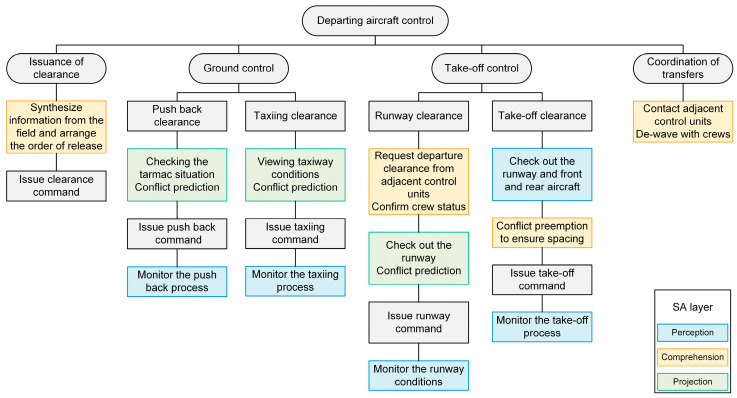
The process of departing aircraft control in the remote tower scenario mainly involves the SA hierarchy. For ease of visualization, fixed processes that usually follow the issuance of instructions, such as the processes of listening to recitations and filling out electronic process sheets, have been omitted.

In order to design SPAM probe questions conforming to the remote tower scenario, we first systematically sort out the control task characteristics and operational processes in the remote tower scenario using the hierarchical task analysis method. Subsequently, we annotate each process according to Endsley’s SA three-level model, i.e., sensing, understanding, and predicting. Considering the example of a departing aircraft, Figure 3 demonstrates that the control process in the remote tower scenario mainly involves the SA hierarchy.

Probe questions are designed according to the content and the corresponding SA level of the remote tower control process. This ensures that the questions can be adapted to the remote tower scenario and fully cover the core elements of the SA. At the same time, the difficulty level of the questions should be maintained such that they fully test the participants’ ability to perceive and integrate key information. Furthermore, the questions should minimize the interference with the current tasks and not exceed the cognitive load of the participants to ensure that the probe questions are naturally integrated with the task scenario. Table 2 shows some of the SPAM probe questions.

We also assess the relevance of all SPAM probe questions to ramp control decisions. For example, it is critical that the participants know the relative positions and distances between an aircraft and other aircraft or ground vehicles on the taxiway. They should also know whether there are any potential conflicts in the current taxiway path and predict possible future cross-collision scenarios. It is important to note that we do not expect participants to actively “count events”; rather, the logic of SPAM is that the controllers with better SA can quickly locate appropriate information sources and, thus, demonstrate a higher accuracy and faster reaction times when answering the SPAM probe questions [44].

Ultimately, the SPAM obtains the SA value by combining the correctness of the participants’ answers to the probe questions and the reaction time as follows:

(3) $${\mathrm{SPAM}}_{\mathrm{score}}=w_{A}\cdot \mathrm{Accuracy}+w_{\mathrm{RT}}\cdot \left(1-RT_{\mathrm{norm}}\right),$$

In Equation (3), Accuracy represents the correctness of the probe question, and $RT_{\mathrm{norm}}$ is the normalized reaction time. In this study, the three levels of probe questions are weighted equally, while the weights for correctness and reaction time are $w_{A}=0.7$ and $w_{\mathrm{RT}}=0.3$, respectively. The accuracy of responses to probe questions was determined by the experimenters, while the SPAM score was calculated by a programmed algorithm.

### 3.3. TPE-LightGBM

The underlying theory of LightGBM is GBDT, which is a boosting method to optimize the predictive performance of a model by iteratively constructing multiple decision trees [32]. The objective function in the GBDT consists of two parts, a loss function and a regularization term, shown as follows:

(4) $$\mathrm{Obj}=\sum_{i=1}^{n}L\left(y_{i},f\left(x_{i}\right)\right)+\sum_{t=1}^{T}\Omega \left(f_{t}\right),$$

In Equation (4), $L\left(y_{i},f\left(x_{i}\right)\right)$ is the loss function, denoting the sample $\left(x_{i}{,y}_{i}\right)$ of the prediction error. The regularization term $\Omega (f_{t})$ controls the complexity of the tree, e.g., by controlling the number of leaf nodes and the depth of splitting, thus effectively preventing overfitting.

Compared with the traditional GBDT, LightGBM is optimized in several aspects, which significantly improves its efficiency and performance. First, the LightGBM discretizes continuous features into a histogram form using a histogram-based decision tree splitting algorithm, which significantly reduces the computational complexity and speeds up model training. Second, LightGBM introduces a leaf-wise growth strategy, where the leaf node with the largest gain is selected for splitting each time. This strategy reduces the loss function more efficiently than the traditional layer-based, i.e., level-wise growth, approach, improving the prediction accuracy of the model. In addition, LightGBM optimizes the samples using the gradient-based one-side sampling (GOSS) method. This method prioritizes the retention of samples with larger gradients, while those with smaller gradients are randomly retained. It reduces the computational effort and maintains the structure of the gradient distribution, thus ensuring the accuracy of the model.

However, LightGBM models cannot fully explore the parameter space when hyperparameters are chosen either manually or empirically, which may cause overfitting or underfitting under certain parameter combinations. Whereas the traditional genetic algorithm process is complex, the random search algorithm performs poorly for a small dataset size [45]. To solve this problem, we introduce the TPE to optimize the parameter selection of LightGBM.

The principle behind the TPE is to model the distribution of the objective function (x), which transforms the hyperparametric optimization problem into a conditional probability problem. Specifically, the TPE divides the sampling points of the objective function into two parts: l(x), the set of parameters with better performance, i.e., those that give a lower value of the objective function, and g(x), the set of parameters with worse performance, i.e., those that produce a higher value of the objective function. These two sets are modeled separately. By estimating P(x|f(x)<τ) and P(x|f(x)≥τ), the TPE uses the Bayes’ theorem to transform the optimization problem into the following form:

(5) $$P\left(x|f\left(x\right)<\tau \right)\propto P\left(f\left(x\right)<\tau |x\right)P\left(x\right),$$

In Equation (5), τ is the performance threshold, which is used to classify the superior and inferior parameters. The TPE utilizes these two probability distributions to construct the generative model, where l(x) is used to guide the sampling of new parameters, and g(x) provides exploratory information so as to focus the sampling near the optimal solution and gradually approach it.

Compared with the traditional Bayesian optimization methods, e.g., Gaussian process-based modeling, the TPE performs more efficiently in high-dimensional parameter spaces, mainly because its model is more suitable for dealing with discrete or conditionally dependent parameters. Furthermore, the TPE adopts nonparametric distribution modeling, e.g., kernel density estimation, which is more adaptable compared to other models as it avoids assumptions on the shape of the objective function.

In this study, we define the search space of hyperparameters using the Optuna framework in Python 3.8 environment and optimize the objective function using the TPE. The performance of each set of hyperparameters during the optimization process is evaluated by 5-fold cross-validation. The root mean square error (RMSE) is used as the objective function to quantify the prediction error of the model and guide the optimization direction of the TPE algorithm. This study is conducted over 100 iterations, with each iteration updating the probabilistic model based on the performance of the current hyperparameters to continuously approximate the optimal hyperparameters. Table 3 shows the final hyperparameter values obtained using TPE optimization.

After obtaining the hyperparameters through TPE optimization, the model is retrained using 10-fold cross-validation.

### 3.4. SHAP

The SHAP is an interpretation method based on Shapley values for interpreting the prediction results of complex machine learning models [31]. The principle behind Shapley values is based on the game theory, which is used to measure the marginal contribution of each participant in a cooperative game to the overall payoff. The SHAP is generalized to machine learning, measuring the contribution of each feature to the predicted outcome of the model. Assuming that the set of model features is N, the Shapley value quantifies the contribution of a particular feature i by calculating the effect that its addition to the subset of features S has on the model output, i.e.,

(6) $$\phi_{i}=\sum_{S\subseteq N\setminus \left\{i\right\}}\frac{\left|S\right|!\cdot \left(\left|N\right|-\left|S\right|-1\right)!}{\left|N\right|!}\cdot \left[f\left(S\cup \left\{i\right\}\right)-f\left(S\right)\right],$$

In Equation (6), S is the subset that does not contain feature i, f(S∪{i}) is the predicted value after adding the feature i to subset S, and $\frac{|S|!\cdot \left(|N\right|-\left|S\right|-1)!}{|N|!}$ is the weight, which represents the probability of alignment of a subset of features. The Shapley value considers all possible combinations of features and averages the marginal contributions of the feature i across different subsets of features, thus ensuring the fairness of the computed results.

The Shapley value satisfies three basic properties: efficiency, which indicates that the sum of all feature contributions is equal to the actual output value of the model; symmetry, which indicates that two features with equal contribution to the model output have identical Shapley values; and the zero-contribution property, which indicates that a feature that does not affect the model prediction has a Shapley value of zero. These properties ensure the fairness and interpretability of Shapley values in feature importance assessment and are particularly suitable for interpreting the prediction results of complex machine learning models.

There are many algorithms that can be used to implement SHAP, out of which the Tree SHAP is optimized for tree models, effectively reducing the computational complexity. Specifically, the algorithm recursively calculates the weights assigned to the paths of features in the decision tree, which reduces the computational complexity from an exponential (2^N^) to a polynomial level (TL^2^), where *T* and *L* denote the number of trees and the average number of leaf nodes per tree [46].

We further improve the computational efficiency by introducing GPU acceleration techniques to accelerate the computation of SHAP values. Specifically, the GPU Tree SHAP technique utilizes the multi-core parallel computing capability of GPUs to simultaneously compute the feature contributions of multiple paths in a single tree, significantly accelerating the computation of path weights. To calculate the cumulative SHAP values of multiple trees, the GPU Tree SHAP technique can process multiple samples at the same time, which significantly improves the computational efficiency and outperforms the traditional CPU implementation, especially in terms of time complexity and throughput. In addition, this technique reduces the memory bandwidth bottleneck by optimizing the GPU memory model, e.g., using shared memory, which further improves the overall computational efficiency.

**Figure 4:**
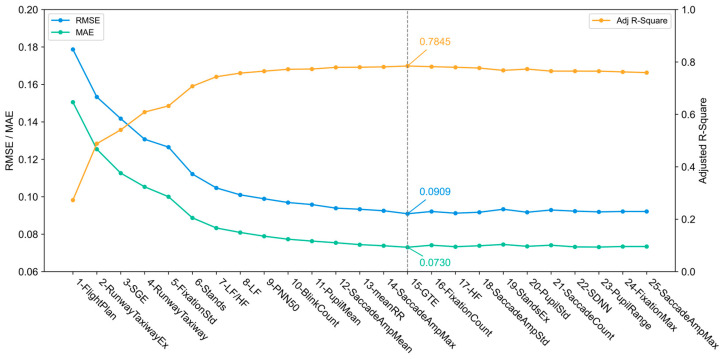
TPE-LightGBM model performance versus addition of features in order of importance.

We use SHAP to rank the importance of the features and gradually add feature variables to the model based on this ranking to calculate the classification performance of TPE-LightGBM. As Figure 4 shows, the performance of the model becomes optimum and stabilizes once the number of features increases to 15. Therefore, the first 15 features are finally selected as input variables for the model to ensure a good balance between performance and computational complexity.

## 4. Results

### 4.1. Prediction Results

We evaluate the prediction performance of the model using RMSE, MAE, and adjusted R-Square, which are defined as follows:

(7) $$\mathrm{RMSE}=\sqrt{\frac{1}{n}\sum_{i=1}^{n}(y_{i}-{\hat{y}}_{i}{)}^{2}},$$

(8) $$\mathrm{MAE}=\frac{1}{n}\sum_{i=1}^{n}|y_{i}-{\hat{y}}_{i}|,$$

(9) $$R^{2}=1-\frac{\sum_{i=1}^{n}(y_{i}-{\hat{y}}_{i}{)}^{2}}{\sum_{i=1}^{n}(y_{i}-\overset{-}{y}{)}^{2}},$$

(10) $$R_{\mathrm{adj}}^{2}=1-\left(\frac{\left(1-R^{2}\right)\left(n-1\right)}{n-p-1}\right),$$

In Equations (7)–(10), $y_{i}$ is the true value and ${\hat{y}}_{i}$ is the value predicted by the model.

To further assess the effectiveness of the TPE-LightGBM model in SA prediction, we compare its performance with several representative regression models, including Random Forest, Neural Network, XGBoost, and CatBoost, as well as LightGBM with default parameters. The specific experimental results are presented in Table 4.

It can be observed from the table that the TPE-LightGBM model performs the best among all the compared models, obtaining the smallest RMSE and MAE while achieving the highest adjusted R-Square value. This performance suggests that by combining the first 15 features selected by the SHAP and the hyperparameters optimized by the TPE, the TPE-LightGBM can provide superior performance in the remote tower SA prediction task, with a higher accuracy and stronger fitting capability.

### 4.2. SHAP Results

#### 4.2.1. Explaining Global Feature Selection

**Figure 5:**
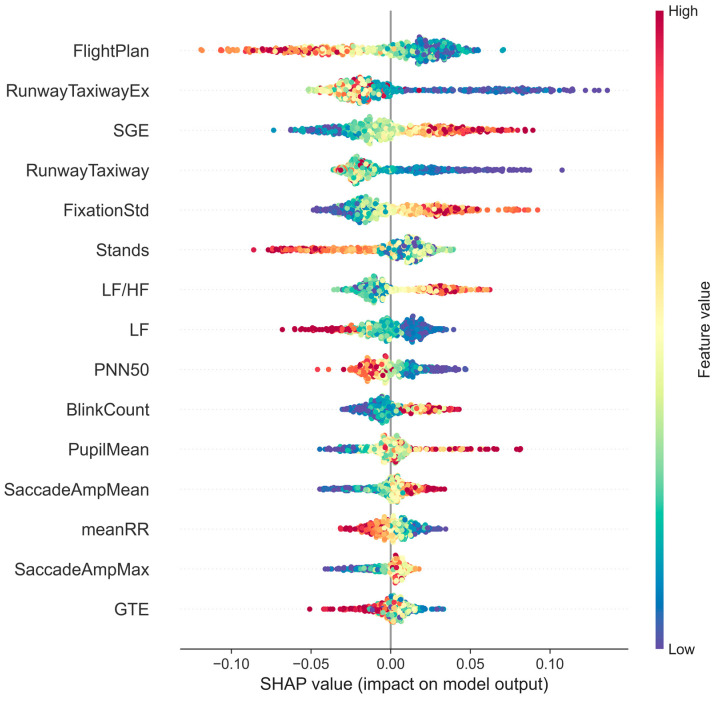
Characteristic swarm map based on SHAP analysis results.

Figure 5 shows the feature density swarm plots, which are generated by the SHAP to visualize the importance of features and feature effects. The horizontal axis of the graph represents the SHAP values, with positive and negative values indicating the positive and negative effects of the feature on the model prediction, respectively. The vertical axis shows a list of the input features sorted according to their importance, with the higher feature position indicating its greater importance to the model output. Furthermore, the color of each point represents the actual magnitude of the feature’s value, which is coded using a color gradient varying from low (blue) to high (red). The horizontal span of the point cloud for each feature shows the range of influence of the feature value on the model output, while its density and shape provide a visual perception of the importance and stability of the corresponding feature.

#### 4.2.2. Explaining the Effect of Features on SA

**Figure 6:**
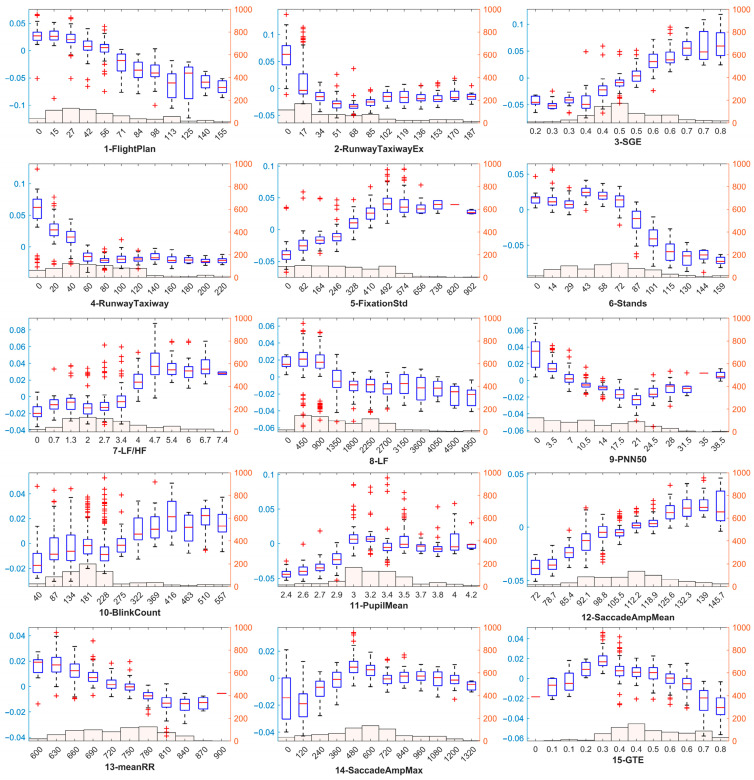
Main effects of SHAP and sample distribution of the dataset.

To provide a more visual demonstration of the effect of the variables on SA, we use the method proposed by Zhou et al. to plot the main effects of SHAP in a box plot, as shown in Figure 6. We further plot a histogram of the sample distribution of the dataset, grouping the sample values into 12 intervals. We also calculate the Pearson’s correlation coefficients between the characteristic variables and their SHAP values to indicate the individual contributions of the variable value sets to the SA [47].

In terms of eye-tracking features related to gaze behavior, all four AOI region visit count features are negatively correlated with the SA. These include the most important feature 1-FligtPlan (r = −0.851, *p* = 0), the second most important feature 2-RunwayTaxiwayEx (r = −0.636, *p* = 0), feature 4-RunwayTaxiway (r = −0.706, *p* = 0) and feature 6-Stands (r = −0.623, *p* = 0). In addition, feature 3-SGE (r = 0.904, *p* = 0) and feature 15-GTE (r = −0.677, *p* = 0) are correlated with entropy values, indicating that the participants with a higher entropy of resting gaze and a lower entropy of gaze have a higher SA. However, very low values of leap gaze entropy (below 0.2) also decrease the SA. The only direct gaze feature, 5-FixationStd (r = 0.894, *p* = 0), indicates that the higher the standard deviation of gaze duration, the higher the SA of the participants. The saccade-related features, 12-SaccadeAmpMean (r = 0.8378, *p* = 0) and 14-SaccadeAmpMax (r = 0.2021, *p* = 0), are both positively correlated with the SA, but there is no significant effect of the maximum distance of a single eye jump exceeding 600 pixels on the SA. Out of the eyeball-related features, the correlation value of 10-BlinkCount (r = 0.3043, *p* = 0) reflects that participants with a high number of blinks also have a higher SA. While another feature, 11-PupilMean (r = 0.126, *p* = 0.698), negatively affects the SA for a small pupil diameter, it is the only feature that does not significantly correlate with its SHAP value.

In terms of HRV, the feature with the highest significance level, 7-LF/HF (r = 0.6094, *p* = 0), indicates that the higher the ratio of low-frequency power to high-frequency power in the participant, the higher the SA. On the other hand, the remaining HRV features, including feature 8-LF (r = −0.6498, *p* = 0), feature 9-PNN50 (r = −0.846, *p* = 0) and feature 13-meanRR (r = −0.870, *p* = 0), are all negatively correlated with SA values.

#### 4.2.3. Explaining Individual Instances

**Figure 7:**
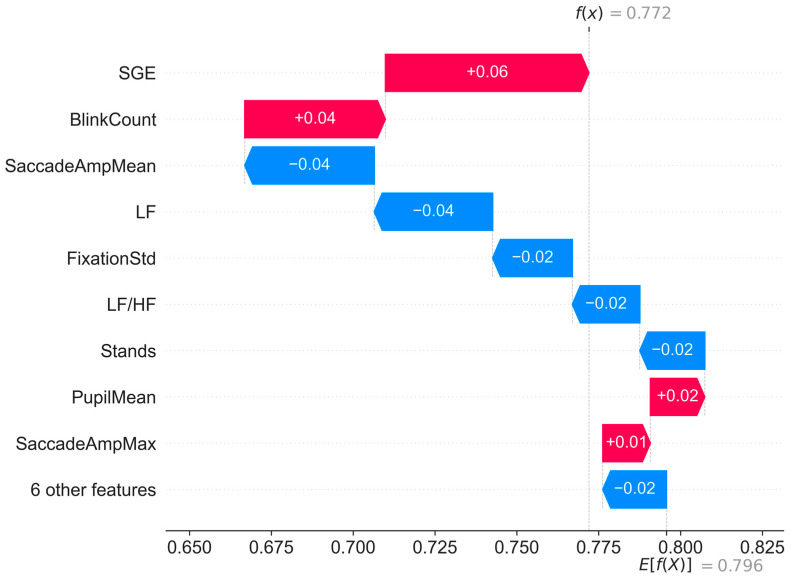
Single-sample interpreted plot based on SHAP values (sample 003).

Figure 7 shows a single-sample explanatory plot that is generated based on the SHAP values. It demonstrates the contribution of the LightGBM model to the individual features in the sample 003 predictions. The horizontal axis represents the range of predicted values, the length of the arrows shows the degree of influence of the feature on the model output, and the color and direction of the arrows indicate the positivity or negativity of the influence. On the bottom-right corner, E[(X)]=0.796 shows the baseline-predicted value of the model, i.e., the global SHAP mean, whereas f(x)=0.772 on the upper-left corner is the final predicted value of the current sample.

In this sample, SGE, BlinkCount, SaccadeAmpMean and PupilMean contribute positively to the predicted value, shown by the red color, while SaccadeAmpMean, LF, FixationStd, LF/HF, stands and six other features decrease the predicted value, shown by the blue color. The contributions of the positive and negative features are superimposed to obtain an overall predicted value of 0.751, while the true SA is 0.739. However, the importance rankings of the features in individual instances may be different from the global importance rankings because the global importance is usually obtained by analyzing all the samples together.

## 5. Discussion

### 5.1. Predicting SA

In this study, we predict the SA levels of controllers in a remote tower environment by using a modified LightGBM model with eye-tracking and HRV data as input.

First, we use the SPAM method to obtain the labeled dataset used for training the model, which combines direct measures of SA with the participants’ correct response rates and reaction times. The remote tower control task scenarios usually span a long duration and involve work at a relatively more sedate pace. However, this real-time measurement method based on probe questions may still interfere with the continuity and naturalness of the control task to some extent [48]. A promising optimization direction involves the development of more indirect SA assessment methods in conjunction with task performance indicators that are directly related to the SA. These indicators can reflect the participants’ SA levels at the operational behavior outcome level and avoid the interference of frequent probe insertion problems on the task fluency. However, there is a lack of feasible and systematic methods that can quantitatively assess task performance in remote tower scenarios, especially for ramp control tasks. The absence of such methods limits the further development of SA measurement based on behavioral outcomes.

Second, we determine the hyperparameters of the LightGBM model using the TPE, which significantly increases the model training time. As an example, on a consumer-grade PC with a 3.0 GHz Intel Core i9 and NVIDIA RTX 4090 24 GB, the training time increased from 8.254 s to 21.349 s with 720 sets of data. However, the optimized model shows stable results in 10-fold cross-validation; therefore, the hyperparameters obtained using the TPE can be used directly in subsequent prediction tasks to avoid repeating the time-consuming optimization process. Meanwhile, the optimized hyperparameters shown in Table 3 significantly improve the prediction accuracy of the model. As accuracy is a key indicator of model performance, the increased training time cost of the TPE optimization process is acceptable.

In addition, the proposed model has non-invasive and real-time characteristics and does not involve features directly related to SA measurements. In the future, an integrated detection system can be built based on this model for real-time monitoring of the SA status of controllers in remote tower scenarios and subsequently providing appropriate interventions as needed. In addition, by expanding the dataset size and introducing further modal physiological features, e.g., EEG signals, skin electrical responses, etc., the predictive performance and generalization ability of the model are expected to be further improved. This can provide more comprehensive support for SA assessment in complex task scenarios. Beyond SA assessment, the model also provides valuable insights into air traffic controllers’ workload by identifying physiological and behavioral patterns related to SA. This lays the foundation for future workload-aware control strategies, where real-time ATC workload monitoring can enhance operational safety and efficiency.

### 5.2. Explaining SA Prediction

In this study, we use SHAP for model input feature selection while explaining the effect of features on the SA and individual prediction examples.

First, a total of 15 features are selected as input variables for the best model based on SHAP’s ranking of feature importance. These features include 11 eye-tracking-related features and four HRV features. These features can reflect the participants’ attentional distribution, visual behavioral patterns, and cognitive regulation in performing the task, as well as revealing their physiological arousal levels and autonomic activity properties [49].

Second, the effect of variables on SA is studied. Eye-tracking-related features are categorized into three main types, gaze, eyeballs, and saccade, with gaze having the highest level of importance. The more often each AOI region is gazed upon, the lower the SA of the participants. This may be because the high-frequency visiting behavior is usually caused by a lack of clear goal guidance in the task. Consequently, the participants randomly switch between different AOI regions rather than following the task logically and acquire the information in an organized manner, as evidenced by the negative relationship between the leap gaze entropy and the SA. The high number of visits may also be related to an overreliance on visual cues. In some tasks, the participants may compensate for a lack of understanding of the context of the task or lapses in their memory by repeatedly confirming the visual information. It is important to note that a lower number of visits does not imply a lower number of gaze points in the AOI region. On the contrary, the number of gaze points in critical regions tends to show a positive correlation with the SA [50].

Meanwhile, controllers with a good SA have a wider distribution of gaze between multiple task areas and a more flexible and variable allocation of gaze time, as indicated by a higher standard deviation of static gaze entropy and gaze time. This gaze pattern contributes to a more comprehensive perception of the complex task environment and the ability to integrate information more effectively, suggesting that the controllers can flexibly allocate their attention according to task demands. In addition, Das and Maiti noted that when an operator exhibits both high gaze entropy and high gaze capacity, it indicates their ability to process a large amount of visual information, indicating constant vigilance over multiple areas [51]. For example, when approaching aircraft are being monitored, gazing at the runway entrance for an extended period of time helps to accurately obtain relevant information and respond in a timely manner. The SA is also higher for participants that exhibit a higher number of blinks in the eyeball-related traits, which is different from the finding in Simon et al. that the blink rate decreases with increasing mental workload [52]. This may be because the controller’s task load in a remote tower environment may be at a moderate level rather than a high-stress state. At the same time, blinking behavior may also mark “switching points” in the cognitive state; i.e., controllers may blink briefly between integrating the information or completing thought processes.

On the other hand, the mean pupil diameter and SA have a more complex relationship. Although a smaller pupil diameter negatively affects the SA, its overall correlation is weak, and it is not significantly correlated with the SHAP values. This may be because the pupil diameter is also influenced by multiple factors, such as individual physiological characteristics, light intensity of the task environment, and the participant’s emotional state [53]. In terms of saccade, the participants can cover a wider range of visual areas when the eye jump amplitude is higher. This behavior results in the acquisition of a higher amount of task-relevant information, which is also consistent with the findings on gaze behavior [54]. In summary, controllers in remote tower environments with a high SA seem to prefer more sequential and extensive visual patterns and flexible gaze duration strategies while minimizing information area switching.

In terms of HRV, the feature with the highest importance is the ratio of low-frequency power to high-frequency power. It is widely regarded as an important measure of autonomic nervous system homeostasis. Its higher values usually indicate a predominance of sympathetic activity, which is normally associated with higher levels of physiological arousal and the ability to schedule cognitive resources. In addition, the negative correlation of PNN50 with SA suggests that when there is an excessive vagal activity; the participants may be in a state of over-relaxation, leading to decreased task engagement and inattention. Meanwhile, the RR interval values that are negatively correlated with the SA usually reflect higher levels of physiological arousal when they are lower [55]. These results are all consistent with the findings of the low-frequency power to high-frequency power ratio, further supporting the positive effect of higher levels of physiological arousal on elevated SA. However, the negative correlation between the low-frequency power and SA suggests that while a certain level of sympathetic activation contributes to the elevated SA, an excessively high high-frequency power may reflect an excessively high cognitive load, causing distraction and decreased SA levels.

Last, we also explain the contribution of individual features in individual prediction instances. In the prediction for sample 003, the features of resting gaze entropy, number of blinks, average eye jump amplitude, and low-frequency power contribute the most to the predicted values. Note that the feature importance ranking in a single sample may differ from the global feature importance, because the latter is derived based on all the samples combined. On the other hand, a single instance reflects the localized impact of a particular feature in that instance and allows for a more fine-grained analysis of the predictive mechanisms in a particular instance.

## 6. Conclusions

In this study, we used eye-tracking data and HRV to predict the SA of remote tower controllers. We designed probe questions based on the SA hierarchy that corresponded to the control task flow in the remote tower scenario and used to obtain a labeled dataset using the SPAM method for the direct measurement of SA. Subsequently, the dataset was trained using the LightGBM model with TPE-optimized hyperparameters, and the input feature variables of the model were selected using SHAP. The TPE-optimized-LightGBM model using 15 feature variables exhibited excellent performance compared with other selected machine learning models, obtaining an RMSE = 0.0909, MAE = 0.0730, and adjusted R-Square = 0.7845. In addition, we used SHAP to provide interpretability to the machine learning model, describing the specific contributions of different feature variables to the SA prediction results. This study proposes an innovative and effective solution for assessing and predicting controllers’ SA in remote tower environments, providing a theoretical basis for understanding the effect of physiological states on SA while also contributing to workload-aware decision-support strategies in air traffic control operations. Furthermore, by offering an interpretable SA monitoring method, this research lays the groundwork for future integration into remote tower systems, aiming to optimize information presentation and workload management for controllers, ultimately enhancing operational efficiency and safety.

Our future work will extend the limitations of the current study. Although the display characteristics of remote tower virtualization tools are suitable for experiments involving simulated environments, some factors present in real scenarios still need to be included, e.g., changes in lighting conditions, delays in communication systems, etc. Therefore, real working scenario characteristics must be incorporated for validation in future studies. Although our study focuses on routine remote tower operations, future research could explore how controllers’ SA adapts to unexpected disruptions, such as temporary signal degradation or runway incursions. Investigating these factors could provide further insights into the resilience of remote tower operations under non-standard conditions. In addition, future experimental designs should involve increased participation of controllers with extensive real-world control experience and use a higher number of participants to ensure the external validity of the findings and the reliability of the statistical analyses.

## Figures and Tables

**Table 1:** Input features to the model.

Number	Feature	Unit	Explanation
1	PupilRange	mm	Overall variation range of pupil diameter during the task
2	PupilMean	mm	Mean pupil diameter
3	PupilStd	mm	Degree of dispersion of pupil diameter
4	BlinkCount	-	Total number of blinks
5	SaccadeCount	-	Number of rapid eye movements between gaze points
6	SaccadeAmpMean	pixels	Mean distance between gaze points during eye jumps
7	SaccadeAmpStd	pixels	Degree of amplitude fluctuation of eye jumps
8	SaccadeAmpMax	pixels	Maximum distance of a single eye jump
9	FixationCount	-	Total number of gaze points
10	FixationMean	ms	Average duration of a single gaze point
11	FixationStd	ms	Degree of fluctuation in gaze time
12	FixationMax	ms	Maximum duration of a single gaze
13	RunwayTaxiwayEx	-	Visits to runway and taxiway exteriors (RTE)
14	StandsEx	-	Visits to the stands exterior (STE)
15	RunwayTaxiway	-	Visits to runways and taxiway (RT)
16	Stands	-	Visits to the stands (ST)
17	FlightPlan	-	Visits to the flight plan (FP)
18	SGE	-	Spatial uncertainty in the gaze points distribution
19	GTE	-	Uncertainty in transition patterns between gaze points
20	LF	ms^2^	Low-frequency components of HRV
21	HF	ms^2^	High-frequency components of HRV
22	LF/HF	-	Ratio of low-frequency to high-frequency power
23	MeanRR	ms	Mean of adjacent heartbeat intervals
24	SDNN	ms	Standard deviation of the heartbeat interval
25	PNN50	%	Percentage of adjacent heartbeats with interval difference greater than 50 ms

**Table 2:** Some SPAM probe questions.

Layer	Examples of Questions
Perception	How many aircraft are currently taxiing on taxiway C6?
	Is there any traffic congestion on Taxiway E8?
Comprehension	Does the taxiing aircraft maintain a safe operating distance from other aircrafts?
	Is the current stands occupancy rate over 40%?
Projection	Is there a potential conflict on taxiway C2?
	Flight CZ9012 is expected to take off in how many minutes?

**Table 3:** Hyperparameters for TPE acquisition.

Hyperparameters	Default Value	TPE Optimized Values	Role
num_leaves	31	182	Maximum number of leaf nodes in each decision tree to control the model complexity
learning_rate	0.1	0.0316	The step-size of each increment is used to control the speed of model convergence
feature_fraction	-	0.5200	Proportion of training features used for controlling feature sampling
lambda_l1	-	0.1761	L1 regularization parameter to control sparsity and prevent overfitting
lambda_l2	-	2.2683	L2 regularization parameters to control model complexity
min_data_in_leaf	20	20	Minimum number of samples required per leaf node to control the minimum sample size of the leaf node

**Table 4:** SA prediction performance of selected regression models.

Model	RMSE	MAE	Adjusted R-Square
Random Forest	0.0953	0.0768	0.7654
Multilayer Perceptron	0.0945	0.0760	0.7701
XGBoost	0.0928	0.0745	0.7802
CatBoost	0.0915	0.0738	0.7820
LightGBM	0.0948	0.0755	0.7785
TPE-LightGBM	0.0909	0.0730	0.7845

## Data Availability

The data generated and analyzed in this study are not publicly available due to confidentiality agreements and privacy protection concerns for the participants. For requests to access the dataset, please contact the corresponding author at lrh27500@foxmail.com.

## Abbreviations

The following abbreviations are used in this manuscript:

SA	Multidisciplinary Digital Publishing Institute
ET	eye-tracking
HRV	heart rate variability
SPAM	scenario presentation assessment method
SHAP	the SHapley Additive exPlanations
ICAO	the International Civil Aviation Organization
SAGAT	SA global assessment technique
SALSA	SA of en-route air traffic controllers in the context of automation
EEG	Electroencephalogram
ECG	Electrocardiogram
SART	the SA rating technique
TPE-LightGBM	LightGBM model optimized by Tree-structured Parzen Estimator
TPE	the Tree-structured Parzen Estimator
GBDT	the gradient boosting decision tree
CAFUC	the Air Traffic Management College of the Civil Aviation Flight University of China
CAAC	the Civil Aviation Administration of China
AOI	the Area of Interest
FFT	the fast Fourier transform
GOSS	the gradient-based one-side sampling
